# MiR-509-3p promotes gastric cancer development by activating FOXM1-mediated p38/MK2 pathway

**DOI:** 10.17305/bb.2024.11104

**Published:** 2024-09-07

**Authors:** Nan Jiang, Jiawei Kang, Yi Ding, Munire Shataer, Liangying Ma, Tayier Tuersong

**Affiliations:** 1 Department of Clinical Medicine, Xinjiang Medical University, Ürümqi, China; 2 Department of Histology and Embryology, Basic Medical College of Xinjiang Medical University, Ürümqi, China; 3 Department of Pharmacy, Xinjiang Key Laboratory of Neurological Diseases, Xinjiang Clinical Research Center for Nervous System Diseases, Second Affiliated Hospital of Xinjiang Medical University, Ürümqi, China

**Keywords:** miR-509-3p, gastric cancer, FOXM1, p38/MK2 pathway

## Abstract

Gastric cancer (GC), a malignant tumor, is highly prevalent, particularly in Asia. miR-509-3p plays a crucial role in regulating tumorigenesis, but its mechanism in GC remains unclear. Potential targets of miR-509-3p were identified through database analyses (miRWalk, TargetScan, ENCORI, and TCGA). The binding site between miR-509-3p and forkhead box protein M1 (FOXM1) was confirmed using a dual-luciferase assay. CCK-8, EdU, Transwell, wound healing assays, flow cytometry, and Western blot analysis were employed to examine changes in proliferation, migration, invasion, apoptosis, FOXM1, and the p38 MAPK (p38)/MAPK-activated protein kinase 2 (MK2) pathway in GC cells (MNK-45 and HGC-27) after miR-509-3p overexpression or knockdown, FOXM1 overexpression, and application of the p38 pathway agonist Anisomycin. The size and weight of subcutaneous xenografts were measured, and the effects of miR-509-3p overexpression were analyzed through histopathological staining (Tunel immunofluorescence, HE staining, Ki67, and FOXM1 immunohistochemistry). The results showed that overexpression of miR-509-3p suppressed proliferation, migration, and invasion, while accelerating apoptosis. Knockdown of miR-509-3p promoted malignant progression. miR-509-3p inhibited GC by regulating FOXM1-mediated p38/MK2 pathway activation, and miR-509-3p mimics restrained tumor growth in vivo through this pathway. In conclusion, miR-509-3p suppresses GC malignant progression by regulating FOXM1-mediated p38/MK2 pathway activation.

## Introduction

Gastric cancer (GC) is a malignant tumor and has become a global health problem [1]. The pathogenesis of GC is related to multiple factors (heredity, environment, and lifestyle), and its incidence is high on a global scale. Although the incidence of GC has decreased, the mortality rate among GC patients in China remains high [2, 3]. According to the burden of malignant tumors in China released in 2024, the number of new GC cases ranked fifth among all tumors (an estimated 358,700 new cases), and the number of deaths ranked third (an estimated 260,400 deaths) [4]. At present, surgery, chemotherapy, and radiotherapy remain important treatments for GC, but their effects on prognosis and the quality of life of patients with advanced GC are often unsatisfactory [5]. Clinical treatment still requires improvements to enhance prognosis and prolong survival. Therefore, identifying new therapeutic targets and breakthroughs is essential. With the continuous development of molecular biology and genomics technologies, more tumor-targeted therapies and immunotherapy strategies are being discovered, offering new hope for GC. However, immunotherapy in GC has started late and progressed relatively slowly. The current main strategy for immunotherapy is immune checkpoint inhibitors (ICI). Existing studies suggest that albumin levels and Royal Marsden Hospital (RMH) scores may serve as prognostic biomarkers for cancer patients receiving ICI treatment [6, 7], but side effects, such as hearing loss and hypertransaminasemia have been reported [8, 9]. Therefore, tumor-targeted therapy may be a better option.

Studying the molecular mechanisms of GC is crucial for developing targeted therapies for specific pathways. microRNA (miR) is a small RNA, consisting of fewer than 30 nucleotides, that does not directly translate into protein and is highly conserved. miRs negatively regulate related genes through base complementary pairing [10], participating in essential biological processes [11, 12]. Among these, miR-509-3p has emerged as a regulator of tumorigenesis. Overexpression of miR-509-3p has been shown to suppress glioma cells and induce apoptosis [13]. However, the mechanism of miR-509-3p in GC has not been documented.

p38 can be activated by various stimuli, such as inflammatory cytokines, DNA damage, and oxidative stress. It has multiple cellular functions, including proliferation, differentiation, and migration [14, 15]. Consequently, the role of p38 in cancer has been widely studied. For instance, phycocyanin promotes the apoptosis of pancreatic cancer cells via the p38 pathway [16]. Additionally, p38 is involved in breast and endometrial cancer by facilitating bone metastasis and enhancing cancer cell survival [17]. MAPK-activated protein kinase 2 (MK2) is a known downstream effector of p38 [18]. MK2 regulates proinflammatory factors and responds to DNA damage, promoting tumor growth and invasion [19], but the roles of p38 and MK2 in GC remain unclear.

Therefore, we hypothesized that miR-509-3p and the p38/MK2 pathway are involved in the malignant progression of GC. Based on this hypothesis, we investigated the role of miR-509-3p in GC cells and nude mouse xenograft models, screened potential targets and regulatory pathways of miR-509-3p, and analyzed the effects of this pathway on the biological behaviors of GC, aiming to provide a theoretical basis for molecular-targeted therapy of GC involving miR-509-3p in the future.

## Materials and methods

### Bioinformatics analysis

Potential targets of miR-509-3p were identified using ENCORI (https://rnasysu.com/encori/), miRWalk (http://129.206.7.150/), and TargetScan (https://www.targetscan.org/vert/_80/) databases. The top 100 upregulated genes in the TCGA GC dataset were obtained from the GEPIA2 database (http://gepia2.cancer-pku.cn/). The binding site between miR-509-3p and forkhead box protein M1 (FOXM1) was predicted using the ENCORI data system.

### Cell culture

Human normal gastric mucosal epithelial cells (GES-1) and GC cell lines (AGS, MNK-45, N87, and HGC-27) were sourced from the Cell Bank of the Chinese Academy of Sciences (Shanghai, China). All cells were cultured in RPMI-1640 medium (ORCPM0110B, ORiCells, Shanghai, China) supplemented with 100 U/mL penicillin–streptomycin solution (C0222, Beyotime, Shanghai, China) and 10% fetal bovine serum (C0235, Beyotime). The cells were maintained in a 5% CO_2_ incubator at 37 ^∘^C.

### Cell transfection

FOXM1 overexpression (FOXM1), control (vector), miR-509-3p knockdown (inhibitor), control (inhibitor NC), miR-509-3p overexpression (mimics), and control (mimics NC) were obtained from RiboBio (Guangzhou, China). Cells were transfected using Lipofectamine 3000 (L3000001, Invitrogen, Austin, TX, USA), and all transfections were performed for 48 h.

### qRT-PCR

RNA was extracted using TransZol (ET111-01-V2, TRANS, Beijing, China), and AMV reverse transcriptase (2621, TAKARA, Tokyo, Japan) was used for reverse transcription to obtain cDNA. TB Green FAST qPCR (CN830S, TAKARA) was used for PCR amplification. The relative mRNA levels were calculated using the 2^-ΔΔCt^ method. U6 served as a control.Primer sequences:miR-509-3p: F: 5′-TCTTGCTGTTCCTGCTCCTG-3′; R: 5′-AACACAGGCGCCTCTTCTAC-3′; U6: F: 5′-CTCGCTTCGGCAGCACAT-3′; R: 5′-TTTGCGTGTCATCCTTGCG-3′.

### 2.5 CCK-8 assay

GC cells were digested with trypsin and inoculated at 1×10^ImEquation1^ cells/well with 200-µL culture medium. The cells were incubated for 0, 24, 48, and 72 h, respectively. Then, 20-µL CCK-8 working solution (CA1210, Solarbio) was added, and the mixture was gently shaken. After thorough mixing, the cells were incubated for 2 h. The absorbance was measured at 450 nm using a microplate reader.

### EdU staining

GC cells were seeded at 2×10^ImEquation2^ cells/well and cultured for 24 h. They were then incubated with 10-µM EdU culture medium for 1 h. After fixation with 4% paraformaldehyde, the cells were treated with PBS containing 0.3% Triton X-100 (X100, Sigma-Aldrich) for 10 min and incubated with Click solution for 30 min. DAPI staining solution (D9542, Invitrogen) was used to stain the nuclei for 10 min. After anti-quenching treatment, the cells were observed and photographed under a microscope to analyze positive staining.

### Wound healing assay

GC cells were digested and collected, with the concentration set to 1×10^ImEquation3^ cells/mL. 1 mL of cell suspension was seeded into a 6-well plate, where a horizontal line had been drawn in advance. Once the cells adhered and reached more than 80% confluence, a vertical line was scratched with a sterile 20-µL pipette tip perpendicular to the horizontal line. Floating cells were washed away, and the remaining cells were incubated in 1640 medium containing 2% serum. Scratch healing was observed at 0 and 24 h, and scratch width was measured using ImageJ to calculate the cell migration rate:Cell migration rate ═ (initial scratch width - scratch width after 24 h) / initial scratch width × 100%.

### Transwell

The transwell chamber was placed in a 24-well plate. (50 µL of precooled Matrigel matrix gel was added to the chamber in advance to assess invasiveness.) 100 µL of 1×10^ImEquation4^ cells/mL suspension was transferred to the chamber, and 500 µL of RPMI-1640 medium containing 15% FBS was added to each well in the lower chamber. The plates were incubated for 24 h. The lower chamber cells were stained with 0.1% crystal violet for 30 min. After washing with PBS, the cells were dried and photographed under a high-power microscope to observe the number of invading cells.

### Flow cytometry

GC cells were collected after different treatments. Approximately 1×10^ImEquation5^ cells were suspended in 500 µL of Binding Buffer. Then, 5 µL of Annexin V-FITC and PI were mixed in, and the reaction was allowed to proceed. Samples were transferred to flow cytometry tubes, and apoptosis was detected using flow cytometry (Agilent, Santa Clara, CA, USA). The apoptosis rate was then calculated.

### Construction of subcutaneous tumor mouse model

A tumor model was established using 4-week-old female BALB/c nude mice. All animals were obtained from the Laboratory Animal Center (Chinese Academy of Sciences, Shanghai, China). The mice were randomly divided into two groups: agomiR NC and agomiR, with 5 mice per group. HGC-27 cells stably transfected with mimics NC or mimics were subcutaneously injected (5×10^ImEquation6^ cells/mouse) to establish a xenograft model. Tumor volume (1/2 × length × width^2^, mm^3^) was measured and photographed using a vernier caliper. On the 28th day, the tumors were carefully excised and weighed.

### Tunel staining

Tunel staining was performed using a Tunel kit (C1091, Beyotime Biotechnology) to assess apoptosis in tumor tissues. Tissues were fixed with 4% paraformaldehyde, dehydrated, embedded, and sectioned into 4-µm paraffin sections. The sections were treated with 20 µg/mL DNase-free proteinase K solution for 30 min and incubated with 50 µL of Tunel detection solution (C1086, Beyotime Biotechnology) for 60 min to label apoptotic cells. DAPI solution was added to stain the nuclei for 5 min. Tunel-positive cells were observed under a microscope after sealing with an anti-fade reagent.

### HE staining

Tumor paraffin sections were stained with hematoxylin (C0107, Beyotime, Shanghai, China) for 15 min, differentiated with 1% acidic alcohol for 30 s, and stained with 0.5% eosin (G1100, Solarbio) for 3 min. The slices were then subjected to alcohol gradient dehydration and xylene treatment, followed by sealing with neutral gum (G8590, Solarbio). Tumor morphology in nude mice was observed under a microscope.

### Immunohistochemistry

Paraffin sections of tumor tissue specimens were subjected to conventional dewaxing, and antigen retrieval was performed using microwaves. The sections were placed in 3% H_2_O_2_ for 25 min and washed. Tissues were blocked with 5% bovine serum albumin (V900933, Sigma-Aldrich) for 30 min. Primary antibodies for FOXM1 (ab207298, 1:250, Abcam) and Ki67 (ab16667, 1:200, Abcam) were added and incubated for 90 min. Secondary antibodies (HRP-labeled goat anti-rabbit IgG, 31460, 1:10000, Invitrogen, Shanghai, China) were added and incubated for 20 min. DAB (DA1010, Solarbio) was used for color development, followed by a tap water rinse. Sections were counterstained with Mayer hematoxylin (MHS16, Sigma-Aldrich), sealed with neutral gum, and observed under a microscope.

### Western blot

After the cells and tumor tissue were collected, the samples were fully lysed using RIPA lysis buffer. Protein concentrations were measured using a BCA kit (PC0020, Solebold). Samples from each group were separated by electrophoresis, and the proteins were then transferred to PVDF membranes (YA1700, Solarbio). The membranes were incubated with 5% skimmed milk powder (LP0033B, Solarbio) for 2 h, and primary antibodies for FOXM1 (ab207298, 1:1000, Abcam), p38 (ab170099, 1:1000, Abcam), p-p38 (ab195049, 1:1000, Abcam), MK2 (ab32567, 1:1000, Abcam), p-MK2 (ab278705, 1:1000, Abcam), and GAPDH (TA-08, 1:1000, ZSGB-BIO, Beijing, China) were applied. The membranes were incubated at 4 ^∘^C overnight. The next day, the membranes were incubated with a secondary antibody (1:20000) for 1 hour. After washing the membranes five times with the TBST buffer, ECL reagent (PE0010, Solarbio) was used for 2–3 min of reaction, and imaging was performed using an automatic chemiluminescence system.

### Ethical statement

This study was approved by the Experimental Animal Ethics Committee of the Second Affiliated Hospital of Xinjiang Medical University.

### Statistical analysis

Data from measurements following a normal distribution were expressed as mean ± standard deviation. Statistical analysis and graph plotting were performed using GraphPad 9.0. Student’s *t*-test was used to compare the differences between two independent groups, and one-way ANOVA was used to compare multiple groups. *P* < 0.05 was considered statistically significant.

## Results

### Overexpression of miR-509-3p inhibits GC progression

**Figure 1.**
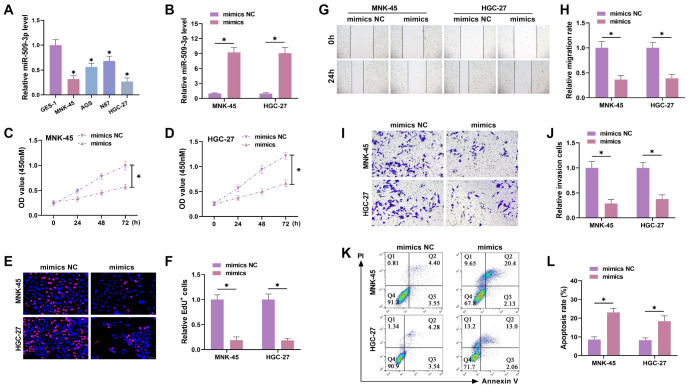
**Overexpression of miR-509-3p suppresses GC progression.** (A) The levels of miR-509-3p in human normal gastric mucosal epithelial cells (GES-1) and GC cell lines (MNK-45, AGS, N87, and HGC-27) were detected by qRT-PCR, after which MNK-45 and HGC-27 cells were selected for further study; (B) MiR-509-3p mimics were transfected into GC cells, and transfection efficiency was tested by qRT-PCR; (C and D) CCK-8 assay tested proliferation under different treatment conditions; overexpression of miR-509-3p suppressed proliferation; (E and F) EdU assay tested the proportion of positive cells and evaluated cell proliferation; overexpression of miR-509-3p inhibited proliferation; (G and H) Wound healing assay tested cell migration; miR-509-3p overexpression suppressed migration; (I and J) Transwell assay tested the number of cells invading the lower chamber; miR-509-3p overexpression suppressed invasion; (K and L) Flow cytometry tested apoptosis levels; miR-509-3p overexpression promoted apoptosis. GC: Gastric cancer. ^*^P < 0.05.

We used qRT-PCR to assess miR-509-3p levels in different GC cell lines. miR-509-3p expression was lowest in MNK-45 and HGC-27 cells, which were selected for follow-up tests (Figure 1A). We overexpressed miR-509-3p and confirmed its efficiency. miR-509-3p was effectively overexpressed (Figure 1B), allowing for subsequent experiments. Overexpression of miR-509-3p significantly reduced proliferation (Figure 1C and 1D), decreased the number of positive cells (Figure 1E and 1F), lowered the migration rate (Figure 1G and 1H), and reduced the invasion rate (Figure 1I and 1J). Additionally, flow cytometry showed a significant increase in apoptosis (Figure 1K and 1L), indicating that miR-509-3p overexpression can reduce proliferation, migration, and invasion while promoting apoptosis.

### Knockdown miR-509-3p promotes GC progression

**Figure 2.**
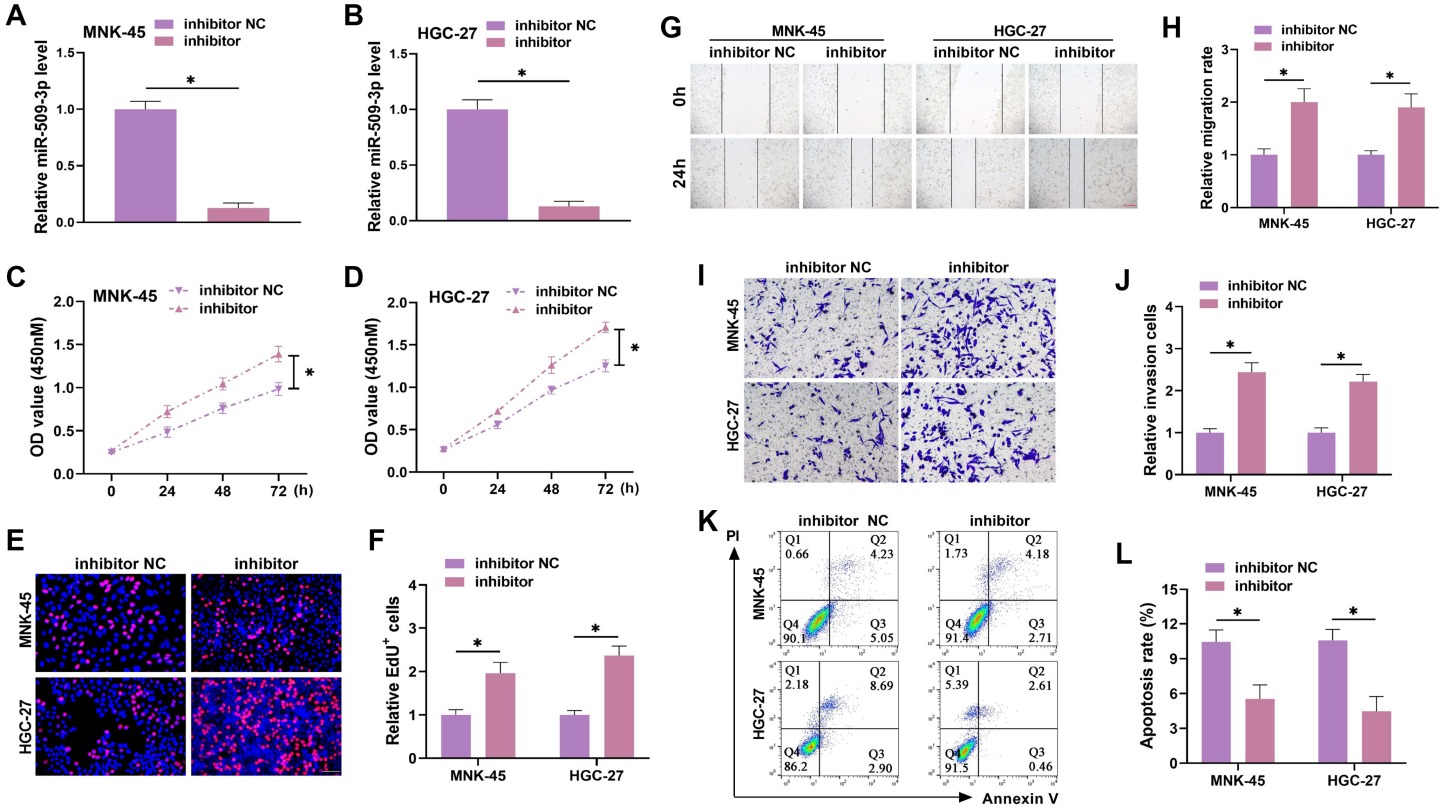
**Knockdown of miR-509-3p promotes GC progression.** (A and B) The miR-509-3p inhibitor was transfected into MNK-45 and HGC-27 cells, and the efficiency was tested by qRT-PCR; (C and D) CCK-8 assays were used to evaluate cell proliferation under different treatment conditions. Knockdown of miR-509-3p promoted cell proliferation; (E and F) EdU assays were used to detect the proportion of positive cells and assess cell proliferation. miR-509-3p knockdown increased the proliferation of GC cells; (G and H) Wound healing assays were used to evaluate cell migration. Knockdown of miR-509-3p enhanced migration; (I and J) Transwell assays were used to measure the number of cells invading the lower chamber. Knockdown of miR-509-3p increased GC cell invasion; (K and L) Flow cytometry was used to assess apoptosis levels. Knockdown of miR-509-3p reduced apoptosis. GC: Gastric cancer. ^*^P < 0.05.

To further verify the role of miR-509-3p, we knocked down miR-509-3p and confirmed knockdown efficiency using qRT-PCR. miR-509-3p was effectively knocked down (Figure 2A and 2B), allowing for further experiments. Knockdown of miR-509-3p significantly increased proliferation (Figure 2C and 2D), and the number of positive cells also significantly increased (Figure 2E and 2F). The wound healing assay revealed that miR-509-3p knockdown significantly increased the migration rate (Figure 2G and 2H). Transwell assays showed that the invasion rate was also significantly raised after miR-509-3p knockdown (Figure 2I and 2J). Furthermore, flow cytometry results showed a marked reduction in the apoptosis rate of GC cells (Figure 2K and 2L), indicating that miR-509-3p knockdown enhances GC progression, making it a key regulator of the malignant behavior of these cells.

### miR-509-3p targeted regulation of FOXM1

**Figure 3.**
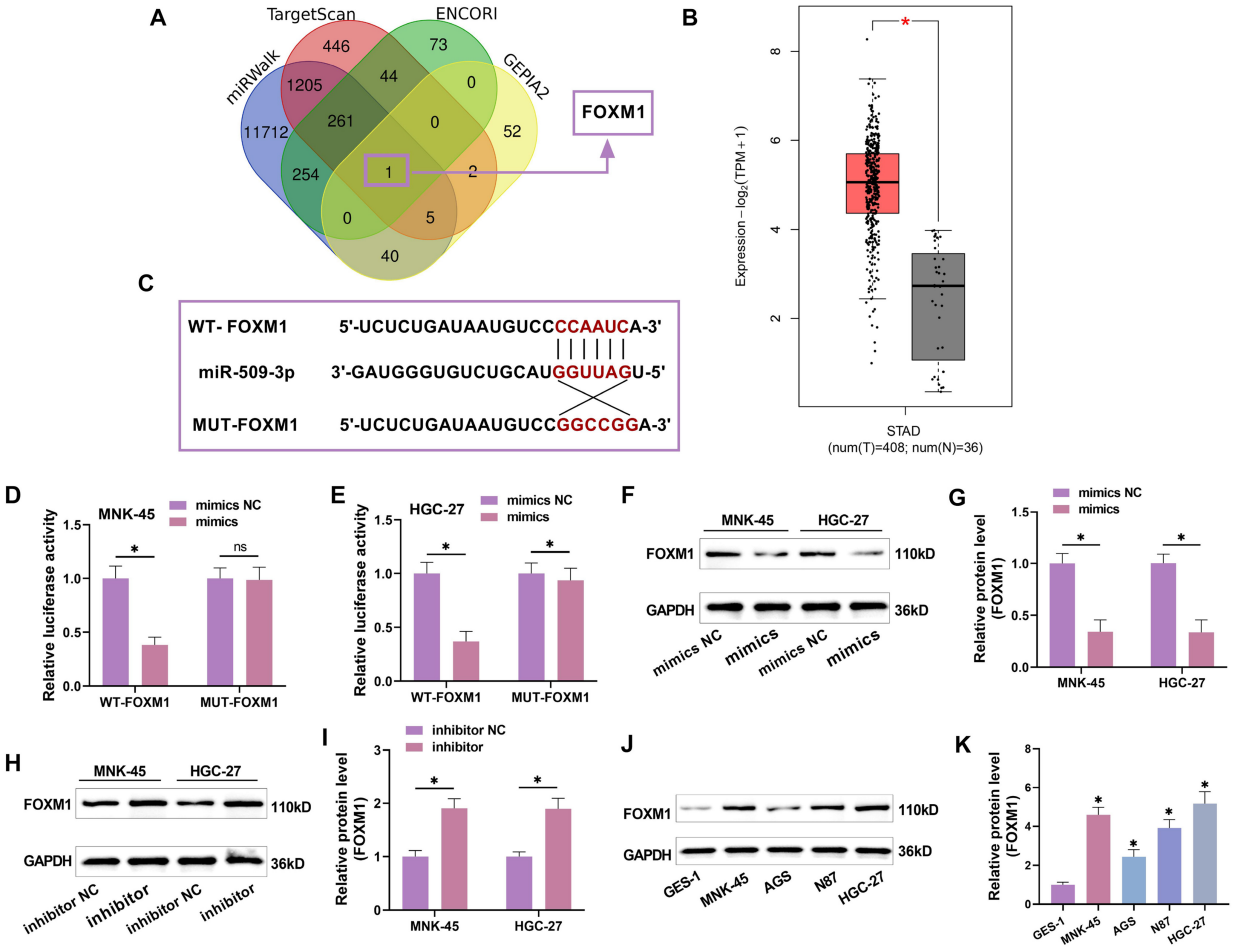
**miR-509-3p regulates FOXM1.** (A) The potential target gene FOXM1 for miR-509-3p was identified through the intersection of miRWalk, TargetScan, ENCORI, and GEPIA2 databases; (B) Analysis of FOXM1 expression in the TCGA GC dataset using the GEPIA2 database showed a significant increase in FOXM1 in GC; (C) The binding site of miR-509-3p to FOXM1 was identified through the ENCORI database, confirming that miR-509-3p can bind to FOXM1; (D and E) WT-FOXM1 and MUT-FOXM1 luciferase reporter plasmids were co-transfected with mimics NC and miR-509-3p mimics into MNK-45 and HGC-27 cells, respectively. It was observed that overexpression of miR-509-3p significantly suppressed the luciferase activity of WT-FOXM1; (F–I) Western blot analysis showed changes in FOXM1 expression after miR-509-3p overexpression or knockdown. FOXM1 protein levels were significantly decreased upon miR-509-3p overexpression and increased when miR-509-3p was knocked down; (J and K) Western blot analysis of FOXM1 levels in GES-1 and GC cell lines confirmed that FOXM1 acts as an oncogene in GC. FOXM1: Forkhead box protein M1; GC: Gastric cancer; GES-1: Gastric mucosal epithelial cells. ^*^P < 0.05.

Bioinformatics analysis identified FOXM1 as a potential target of miR-509-3p (Figure 3A). FOXM1 expression in the GC dataset, analyzed via the GEPIA2 database, was significantly elevated (Figure 3B), suggesting that FOXM1 is an oncogene in GC. Bioinformatics analysis further identified a potential binding site between miR-509-3p and FOXM1 (Figure 3C). Luciferase reporter assays confirmed that miR-509-3p overexpression significantly inhibited the luciferase activity of WT-FOXM1 (Figure 3D and 3E). Western blot analysis showed that FOXM1 protein levels significantly decreased with miR-509-3p overexpression (Figure 3F and 3G) and increased after miR-509-3p knockdown (Figure 3H and 3I), confirming that miR-509-3p directly targets FOXM1. This indicates that FOXM1 is an oncogene in GC, and miR-509-3p can target FOXM1 in GC cells.

### miR-509-3p inhibits GC progression by inhibiting FOXM1

**Figure 4.**
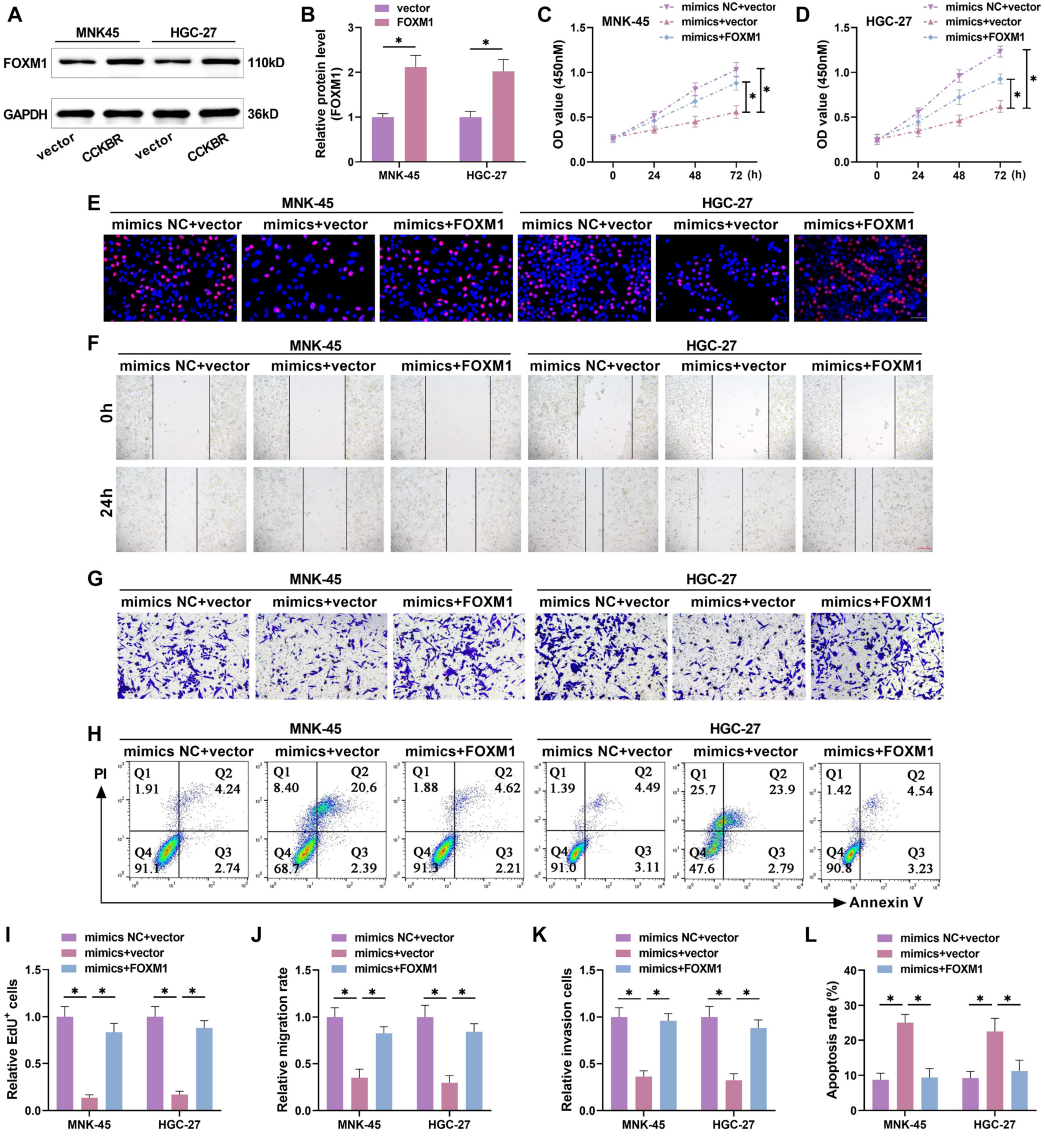
**miR-509-3p inhibits GC progression by inhibiting FOXM1.** (A and B) The FOXM1 mimics was transfected into MNK-45 and HGC-27 cells, and the efficiency was detected by Western blot; (C and D) The miR-509-3p mimics and / or FOXM1 mimics were transfected into GC cells. CCK-8 tested the proliferation after treatment for 24, 48, and 72 h. The proliferation activity was obviously rose after FOXM1 overexpression compared with miR-509-3p overexpression alone; (E and I) EdU detected the proportion of positive cells and evaluated cell proliferation. After FOXM1 overexpression, GC cell proliferation was notably elevated; (F and J) Wound healing assay detected the cell migration. After FOXM1 overexpression, the migration rate of GC cells was markedly increased compared with miR-509-3p overexpression alone; (G and K) Transwell tested the number invading to the lower chamber. After FOXM1 overexpression, the invasion rate of GC cells was obviously increased compared with miR-509-3p overexpression alone; (H and L) Flow cytometry tested the apoptosis level. After overexpression of FOXM1, the apoptosis rate was notably declined. GC: Gastric cancer; FOXM1: Forkhead box protein M1. ^*^P < 0.05.

We further investigated whether miR-509-3p could target FOXM1 to affect GC progression. First, we confirmed the successful overexpression of FOXM1 (Figure 4A and 4B). The experiment was divided into three groups: blank group (mimics NC + vector), miR-509-3p overexpression group (mimics + vector), and miR-509-3p overexpression + FOXM1 overexpression group (mimics + FOXM1). Overexpression of FOXM1 reversed the miR-509-3p-mediated decrease in proliferation (Figure 4C and 4D), EdU-positive cell rate (Figure 4E and 4I), migration rate (Figure 4F and 4J), and invasion rate (Figure 4G and 4K). The miR-509-3p-induced increase in apoptosis was also weakened by FOXM1 overexpression (Figure 4H and 4L), indicating that miR-509-3p suppresses the malignant behavior of GC cells by targeting FOXM1.

### miR-509-3p inhibits GC progression by inhibiting FOXM1-mediated p38/MK2 pathway activation

**Figure 5.**
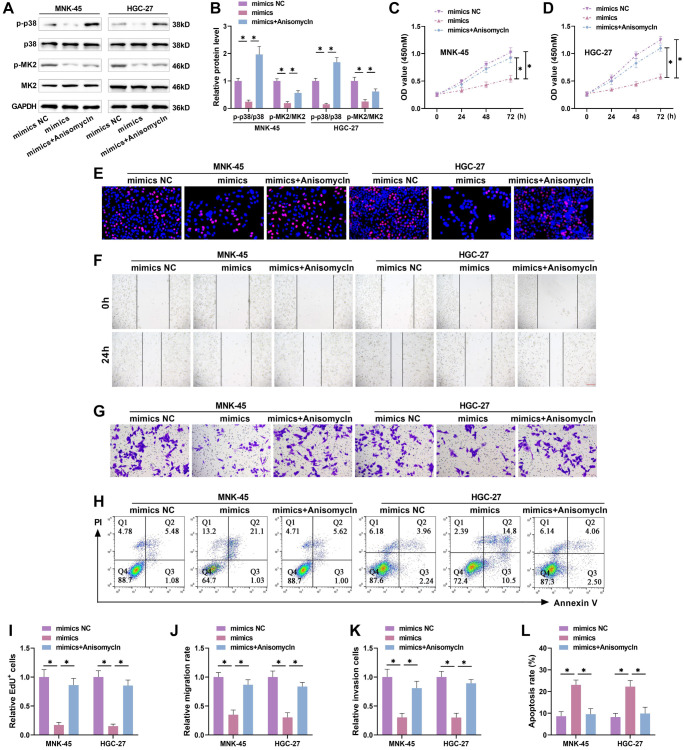
**MiR-509-3p inhibits gastric cancer progression by suppressing p38/MK2 pathway activation.** (A and B) The miR-509-3p overexpression plasmid was transfected into GC cells, followed by treatment with the p38 pathway agonist Anisomycin. The expression of p38/MK2 pathway proteins was assessed by Western blot. After Anisomycin treatment, p-p38 and p-MK2 proteins were upregulated; (C and D) CCK-8 assays were used to evaluate proliferation under different treatment conditions. After Anisomycin treatment, proliferation activity significantly increased compared to miR-509-3p overexpression alone; (E and I) EdU assays detected the proportion of positive cells and assessed cell proliferation. After Anisomycin treatment, GC cell proliferation significantly increased; (F and J) Wound healing assays assessed cell migration. After Anisomycin treatment, the migration rate was significantly increased compared to miR-509-3p overexpression alone; (G and K) Transwell assays measured the number of cells invading the lower chamber. After Anisomycin treatment, the invasion rate was notably elevated; (H and L) Flow cytometry assessed apoptosis levels. After Anisomycin treatment, the apoptosis rate was significantly reduced. GC: Gastric cancer; MK2: MAPK-activated protein kinase 2. ^*^P < 0.05.

**Figure 6.**
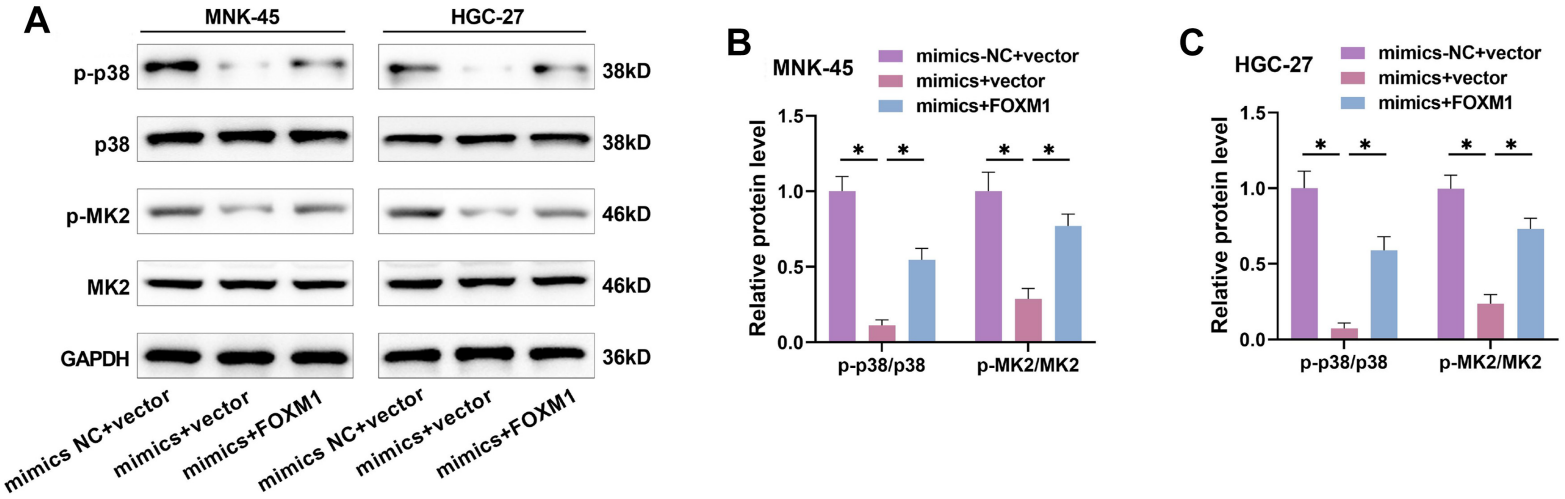
**miR-509-3p regulates the p38/MK2 pathway through FOXM1.** (A–C) MiR-509-3p mimics and/or FOXM1 mimics were transfected into GC cells, and the expression of p38/MK2 pathway proteins was assessed by Western blot. p-p38 and p-MK2 proteins were upregulated after FOXM1 overexpression. FOXM1: Forkhead box protein M1; GC: Gastric cancer; MK2: MAPK-activated protein kinase 2. ^*^P < 0.05.

To verify whether miR-509-3p affects GC progression through the p38/MK2 pathway, we divided the experiment into three groups: blank group (mimics NC), miR-509-3p overexpression group (mimics), and miR-509-3p overexpression + p38 pathway agonist group (mimics + Anisomycin). miR-509-3p overexpression significantly reduced p-p38 and p-MK2 protein levels. When Anisomycin was applied to activate the p38 pathway, p-p38 and p-MK2 levels significantly increased (Figure 5A and 5B), as did proliferation (Figure 5C and 5D), EdU-positive cells (Figure 5E and 5I), migration rate (Figure 5F and 5J), and invasion rate (Figure 5G and 5K), while apoptosis significantly decreased (Figure 5H and 5L). To further confirm whether miR-509-3p targets FOXM1 to regulate the p38/MK2 pathway, the experiment was divided into three groups: blank group (mimics NC + vector), miR-509-3p overexpression group (mimics + vector), and miR-509-3p overexpression + FOXM1 overexpression group (mimics + FOXM1). miR-509-3p overexpression significantly reduced p-p38 and p-MK2 levels, but FOXM1 overexpression weakened this reduction (Figure 6A–6C), indicating that miR-509-3p suppresses the malignant behavior of GC cells by inhibiting FOXM1-mediated p38/MK2 pathway activation.

### miR-509-3p inhibits tumor growth in vivo

**Figure 7.**
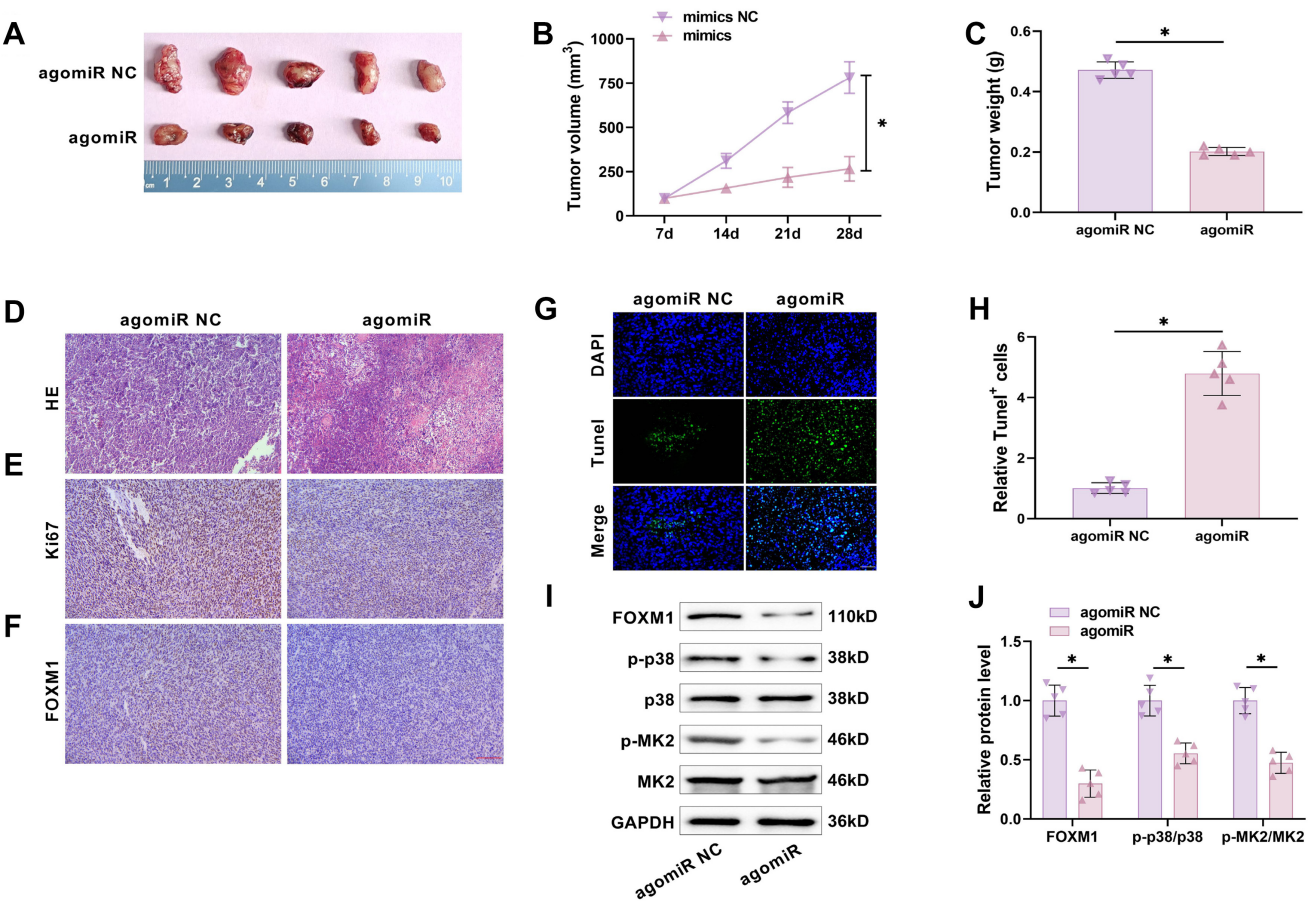
**miR-509-3p inhibits tumor growth in vivo.** (A) Nude mice were subcutaneously injected with HGC-27 cells stably transfected with either NC mimics or miR-509-3p mimics to establish a xenograft tumor model. Tumor volume was measured; (B) Changes in tumor volume during feeding showed that miR-509-3p overexpression reduced tumor volume; (C) Tumor weight in nude mice showed that miR-509-3p overexpression reduced tumor weight; (D) HE staining of tumor tissue revealed that miR-509-3p overexpression increased tumor tissue necrosis; (E and F) Immunohistochemical staining for Ki67 and FOXM1 showed that miR-509-3p overexpression reduced both Ki67 and FOXM1 levels; (G and H) TUNEL assay results indicated that overexpression of miR-509-3p promoted apoptosis; (I and J) Western blot analysis of FOXM1 and p38/MK2 pathway proteins showed that miR-509-3p overexpression notably reduced FOXM1, p-p38, and p-MK2 levels. FOXM1: Forkhead box protein M1; MK2: MAPK-activated protein kinase 2. ^*^P < 0.05.

Given the in vitro results, we further investigated whether miR-509-3p affects GC progression in vivo. A xenograft model was constructed by subcutaneous injection of HGC-27 cells overexpressing miR-509-3p into nude mice. The mice were sacrificed, and tumor size and weight were measured. miR-509-3p overexpression significantly reduced the size and weight of subcutaneous tumors (Figure 7A–7C), increased tumor necrosis (Figure 7D), reduced Ki67 and FOXM1 levels (Figure 7E and 7F), and significantly increased apoptosis (Figure 7G and 7H). Additionally, FOXM1, p-p38, and p-MK2 protein levels were significantly decreased in tumors overexpressing miR-509-3p (Figure 7I and 7J). In summary, miR-509-3p overexpression impedes GC progression.

## Discussion

Cancer statistics for 2022 show that 20 million people will be newly diagnosed with cancer, and nearly ten million will die. The incidence and mortality of GC rank second and third among all malignant tumors worldwide [20]. The early symptoms of GC are not obvious, and patients are usually diagnosed in the middle to late stages. The heterogeneity of GC affects therapeutic outcomes, resulting in a poor prognosis and low survival rates [21]. The low selectivity, poor tolerance, and resistance to current therapeutic drugs make the treatment of GC particularly challenging. As molecular biology research advances, understanding GC at the molecular level will help improve its diagnosis and treatment. miRNA is widely involved in many malignant tumors and is relatively stable, suggesting that miRNA may play an important role in tumor treatment [22].

miR-509-3p has emerged as an important regulator of tumorigenesis and development. miR-509-3p is downregulated in several cancers [23, 24]. For example, miR-509-3p is lowly expressed in renal cell carcinoma, where high expression hinders proliferation and migration [25]. However, studies have also shown that miR-509-3p expression is increased in primary hepatocellular carcinoma and colorectal cancer [26, 27], indicating that miR-509-3p can act as both a tumor suppressor and an oncogene. In this study, we assessed miR-509-3p expression in different GC cell lines using qRT-PCR. The results showed that miR-509-3p expression was lowest in MNK-45 and HGC-27 cells, suggesting that miR-509-3p may function as a tumor suppressor in GC. To investigate the role of miR-509-3p in GC, we overexpressed miR-509-3p, which inhibited GC cell growth. Conversely, miR-509-3p knockdown had the opposite effect, suggesting that miR-509-3p regulates GC progression.

We identified FOXM1 as a potential target gene of miR-509-3p through bioinformatics analysis. The binding sites between miR-509-3p and FOXM1 were further confirmed by bioinformatics. FOXM1 protein levels significantly decreased with miR-509-3p overexpression and increased when miR-509-3p was knocked down. FOXM1 levels were also significantly lower in GC cell lines, further proving that miR-509-3p binds to and targets FOXM1. FOXM1 is a classical transcription factor [28] that promotes tumor metastasis, cell proliferation, differentiation, and invasion [29]. It is associated with nearly all cancer traits [30] and shows gene amplification in breast, liver, lung, pancreatic, cervical cancers, and medulloblastoma, where it is upregulated at the transcriptional level [31–39]. FOXM1 is also closely linked to GC, where overexpression inhibits cell senescence [40], and disruption contributes to GC progression [41, 42]. FOXM1-mediated Wnt/β-catenin pathway activation promotes GC cell proliferation [43], and FOXM1 mediates various other biological processes [44]. However, it is unclear whether miR-509-3p can inhibit GC by targeting FOXM1. In this study, we found that miR-509-3p inhibits GC progression by inhibiting FOXM1.

p38 is a stress-activated protein involved in apoptosis and plays a key role in this process [45, 46]. The role of p38 in cancer is currently under extensive investigation [47]. Knockdown of p38 inhibits esophageal squamous cell carcinoma, indicating that p38 may be a therapeutic target [48]. p38 is notably elevated in colon cancer [49], and its knockdown inhibits tumor progression [50]. Overall, p38 has been shown to act as an anti-tumor factor [47, 51]. Downstream of p38 is MK2, a kinase involved in differentiation, apoptosis, and cell movement [52]. For instance, MK2 can inhibit glioblastoma cells [53], and the p38 MAPK-MK2 pathway may mediate proliferation and invasion in bladder cancer [54]. The p38-MK2-Hsp27 pathway represents a new mechanism for early cancer transmission [55]. We first verified whether miR-509-3p can affect GC progression through the p38/MK2 pathway. miR-509-3p overexpression significantly reduced p-p38 and p-MK2 protein levels. After treatment with p38 pathway agonists, p-p38 and p-MK2 protein levels significantly increased, promoting proliferation, migration, and invasion while inhibiting apoptosis. We then verified whether miR-509-3p regulates the p38/MK2 pathway through FOXM1. Overexpression of FOXM1 alongside miR-509-3p overexpression significantly elevated p-p38 and p-MK2 proteins, indicating that miR-509-3p suppresses GC by inhibiting FOXM1-mediated p38/MK2 pathway activation.

In vivo experiments showed that miR-509-3p overexpression reduced the size and weight of subcutaneous tumors and promoted apoptosis. The pathological structure of tumor tissue improved, and Ki67, FOXM1, p-p38, and p-MK2 levels decreased, further proving that miR-509-3p inhibits GC progression by regulating FOXM1-mediated p38/MK2 pathway activation.

In summary, this study proves that miR-509-3p can inhibit the malignant progression of GC, highlighting the potential of miRNA for tumor treatment with high accuracy. However, drug resistance is a common challenge in this therapy, suggesting that controlling disease progression may require combined treatment approaches. Therefore, combining targeted therapy with chemotherapy and immunotherapy could be the future trend. In addition, the abbreviations of this article are shown in Table 1.

**Table 1:** The list of abbreviations

FOXM1	Forkhead box protein M1
MK2	MAPK activated protein kinase 2
miR	microRNA
GC	Gastric cancer
p38	p38 MAPK

## Conclusion

This study reveals the mechanism of miR-509-3p in GC cell proliferation and tumor growth in vivo. miR-509-3p exerts an anti-GC effect by regulating the FOXM1-mediated p38/MK2 pathway, thereby inhibiting GC progression (Figure S1). This provides a reliable reference for targeted therapy in GC, though some limitations remain. The effectiveness of miR-509-3p in clinical applications needs further evaluation.

## Supplemental data

**Figure S1.**
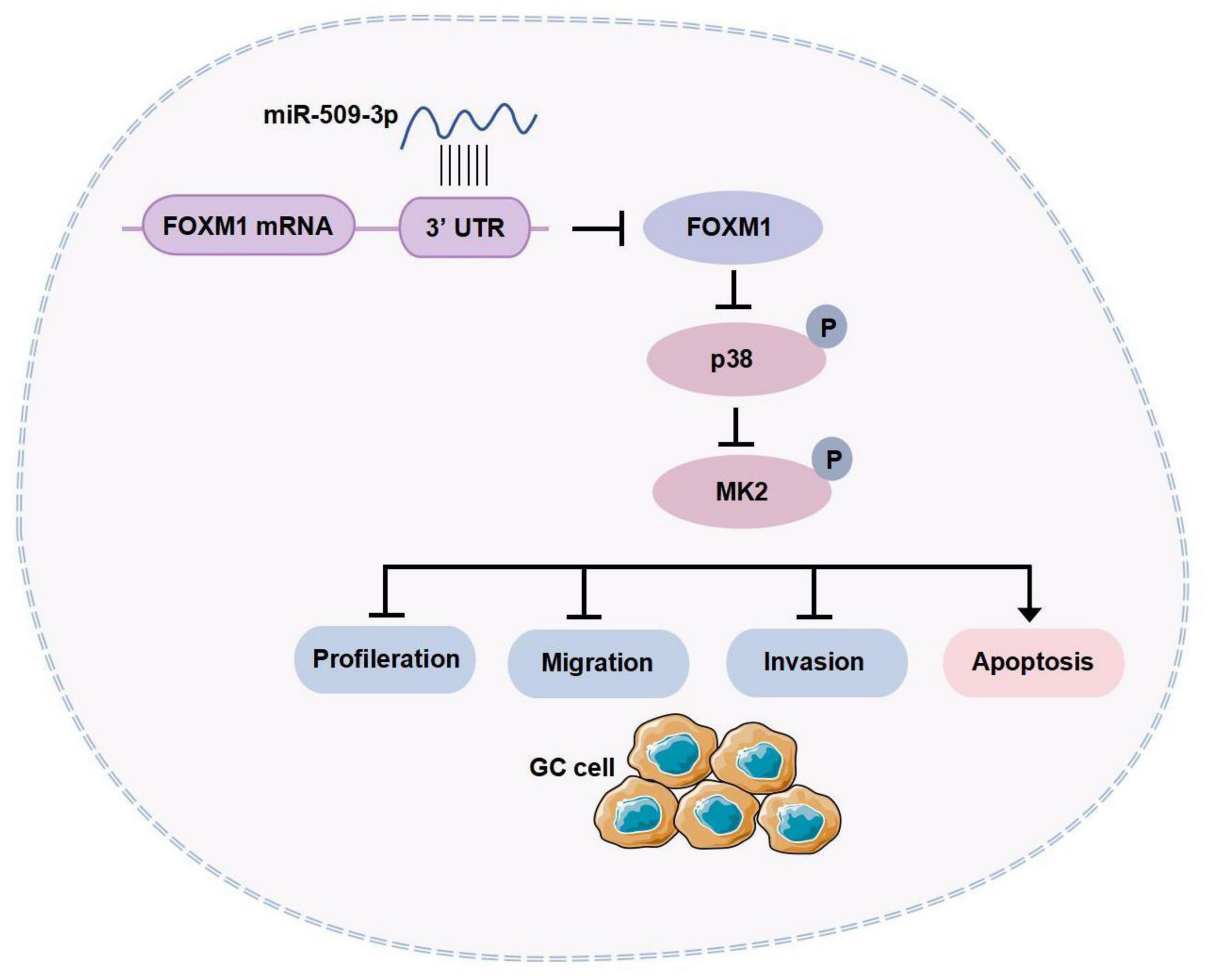
**Graphical Abstract.** miR-509-3p specifically inhibits FOXM1-mediated activation of the p38/MK2 pathway, interfering with proliferation, migration, invasion, and other biological behaviors, thereby impeding the malignant progression of GC. FOXM1: Forkhead box protein M1; GC: Gastric cancer; MK2: MAPK-activated protein kinase 2.

## Data Availability

The data that support the findings of this study are available from the corresponding author, upon request.
